# Design and Analysis of a Continuous Split Typed Needle-Free Injection System for Animal Vaccination

**DOI:** 10.2174/1874120701711010059

**Published:** 2017-06-30

**Authors:** Kai Chen, Min Pan, Tingting Liu

**Affiliations:** School of Mechanical Engineering, Hangzhou Dianzi University, Hangzhou, China

**Keywords:** Needle-free injection, Jet, Animal vaccination, Transdermal injection, Injection pressure

## Abstract

**Background:**

Liquid needle-free injection devices (NFIDs) employ a high-velocity liquid jet to deliver drugs and vaccine through transdermal injection. NFIDs for animal vaccination are more complicated than those used for human beings for their much larger and more flexible power sources, as well as rapid, repetitive and continuous injection features.

**Method:**

In the paper, spring-powered NFID is designed for animal vaccine injection. For convenience, the device is a split into a power source and handheld injector. A mathematical model is proposed to calculate the injection pressure, taking into the account pressure loss and the strain energy loss in the bendable tube due to elastic deformation. An experimental apparatus was build to verify the calculation results.

**Results and Conclusion:**

Under the same system conditions, the calculation results of the dynamic injection pressure match the experimental results. It is found that the bendable tube of the split typed NFID has significant impact on the profile of the injection pressure. The initial peak pressure is less than the initial peak pressure of NFID without bendable tube, and there is occurrence time lag of the peak pressure. The mathematical model is the first attempt to reveal the relationship between the injection pressure and the system variables of split typed NFID.

## 1 INTRODUCTION

Needle-free injection device(NFID) employs a high speed jet to directly puncture the skin for injection [1, 2]. It has been adopted for bulk vaccination of animals [3-8]. It has several advantages: Biological security is greatly increased by elimination of broken needles, needle disposal and needle-stick injuries [6-8]. Vaccination efficiency is enhanced because of high injection speed and lower rate of adverse animal reaction [4-8]. Furthermore, both vaccine absorption rate and antigen dispersion rate are higher than for needle based injection, resulting in a superior immunologic response [3, 6, 8].

Since different animal species have rather different skin properties and the vaccine doses for different animals or different stages of breeding, flexible power must be provided to the needle-free injection system. In addition, the injection location and depth as well as the velocity and/or diameter of the jet stream must be adapted. Mass vaccination of farmed animals often requires continuous injection in order to reduce man power and improve efficiency. Therefore, NFIDs for animal vaccination are often designed with more complicacy than those used for human-beings. Almost all commercially available NFIDs for animal vaccination use compressed air as power source, such as Pulse™ NFID (by Pulse Needle Free Systems Inc., USA), and ARGRO-JET™NFID (by Medical International Technologies Inc., Canada) [6]. Different driving forces can be provided by regulating the air pressure. AcuShot™ NFID (by Acushot Needle Free Inc., Canada) presents a more compact device by applying an air spring as power source, thus eliminating the commonly used air tank [8]. All those animal vaccination NFIDs administer 1.0-2.5 ml injections at 40-220MPa orifice pressures [8].

Although NFIDs for animal vaccination have been adopted in livestock industry, a complete understanding of jet dispersion under various conditions for different animals is rather limited. A lumped parameter which combines jet velocity and nozzle diameter called exit jet power was proposed to describe the performance of jet injectors, and it was found that the percentage of ejected liquid entering the skin increases with increasing jet power [2, 9]. A simple model from the energy balance was also developed by the same researchers [10]. The penetration depth can be formulated as a function of centreline velocity if the jet is considered as a free jet [11]. It is reported that the maximum injection pressure needs to reach a threshold before the jet is able to erode and penetrate into the skin [12]. The authors have developed a quasi-steady physical model to calculate the dynamic injection pressure for a given driving force [13].

There is one common feature of the NFIDs used for mass animal vaccination. The device is often split into power source and handheld injector for convenience of injection, where the driving power is provided either pneumatically or hydraulically through a bendable tube (hose). Part of the driving energy is lost in the bendable tube due to elastic deformation. In this paper, a prototype of a split type NFID powered by a spring with continuous drug injection was designed and implemented. A physical model is presented to calculate the dynamic injection pressure of the current NFID. The measured data are compared with the calculation data.

## 2 SYSTEM DESIGN AND IMPLEMENTATION

**Fig. (1):**
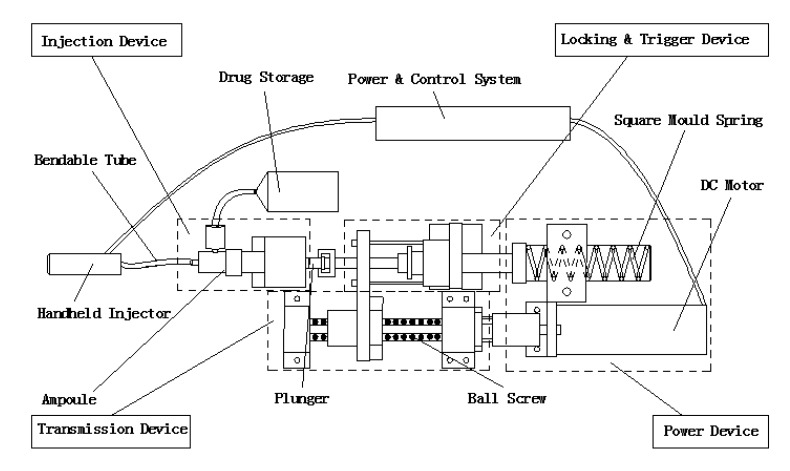
Schematic design of a split-type needle-free system.

**Fig. (2):**
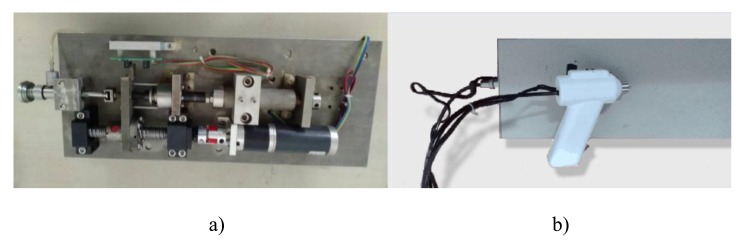
The real implementation of the needle-free injection system: a) internal structure and b) handheld device.

Here, the NFID is composed of a power device, a transmission device, a locking and trigger device, and an injection device, including the drug storage (Fig. **1**). The injector is connected to a handheld device through a bendable tube (hose) (Fig. **2**).

Before the injection, the control system sends a signal to the DC motor to compress the spring by a ball screw assembly and move the plunger backwards. The liquid in the drug storage flows into the ampoule through a one-way check valve. The spring is then locked at a certain location, and the NFID is ready for injection. When the operator triggers from the handheld injector, the controller unlocks the mechanism and releases the spring. The rapidly moving plunger hits the piston in the ampoule, pressing the liquid through the bendable tube and a one-way check valve for injection. After injection, the motor and transmission assembly drives the piston back, and new liquid flows into the ampoule, waiting for the next injection.

## 3 PHYSICAL MODEL AND NUMERICAL CALCULATION

Fig. (**3**) is a general mathematical model of a spring-powered NFID first proposed by Sander and Baker [14] and further improved by Chen *et al.* [13]. The model is able to predict the injection pressure (stagnation pressure of the impinging jet), determine the penetration capability. The elastic resistance and local resistance of bendable tube and local loss of the check valves were not taken into account in the aforementioned mathematical models. However, since energy loss due to elastic deformation in the bendable tube greatly affects the final injection pressure, a modified model is established in this paper taking into these effects.

Based on conservation of mass, it can be obtained that:

(1) $$\rho_{0}\left(A_{p}L+\frac{\pi D_{0}^{2}}{4}L_{1}\right)=\rho \left[A_{p}\left(L-X_{p}\right)+\frac{\pi D_{0}^{2}}{4}L_{1}\right]+\rho_{0}\frac{\pi D_{1}^{2}}{4}\int V_{1}\mathrm{dt}$$

where *ρ*_0 _ and *ρ* are initial and actual fluid density under ambient and actual liquid pressure, respectively. *X_P_* represents the actual displacement of the piston; *L* the initial length of the chamber, and *L_1_* the length of the bendable tube; $A_{p}=\frac{\pi D_{p}^{2}}{4}$ is the cross-section area of the piston, *D_1_* the diameter of the injection orifice, and *D_0_* the diameter of the bendable tube; *V_1_* represents the velocity of liquid stream right coming out of the orifice, where ambient pressure is assumed.

Let P be the actual pressure to ambient pressure and Δ*ρ = ρ − ρ*_0 _ be small .Then the compressibility of the fluid can be expressed by the bulk modulus *B* of the fluid as $\frac{P}{B}=\frac{\Delta \rho }{\rho_{0}}$. Then Equation (1) becomes:

(2) $$0=-A_{P}X_{P}+\frac{P}{B}\left(A_{p}L-A_{p}X_{p}+\frac{\pi D_{0}^{2}}{4}L_{1}\right)+\frac{\pi D_{1}^{2}}{4}\int V_{1}\mathrm{dt}$$

By taking derivative with respect to time on both sides of Equation (2),we obtain:

(3) $$\frac{\mathrm{dP}}{\mathrm{dt}}=\frac{\left(P+B\right)\frac{dX_{p}}{\mathrm{dt}}-\frac{D_{1}^{2}}{D_{p}^{2}}BV_{1}}{L-X_{p}+\frac{D_{0}^{2}}{D_{P}^{2}}L_{1}}$$

For the actual time t, Bernoulli’s equation of pressure balance reads as:

(4) $$P+\frac{1}{2}\rho V_{P}^{2}=\frac{1}{2}\rho_{0}V_{1}^{2}+P_{f}+P_{G}+P_{\mathrm{j1}}+P_{\mathrm{j2}}$$

where $V_{P}=\frac{dX_{p}}{\mathrm{dt}}$ is the velocity of piston (representing the impact of the plunger on the piston, for simplicity), *P_f_* is the frictional drag along pathway, *P*_*j*1_ is the local head loss on the connector between the ampoule chamber and the bendable tube Fig. (**3**). *P*_*j*2_ is the local head loss on the injection orifice, *P_G_* is the strained energy loss of the bendable tube during injection. *P_f_* can be calculated as:

(5) $$P_{f}=\lambda \cdot \frac{\rho L_{1}}{D_{0}}\cdot \frac{V_{0}^{2}}{2}=\frac{\lambda \rho L_{1}}{2D_{0}}\cdot {\left(\frac{D_{1}}{D_{0}}\right)}^{4}\cdot V_{1}^{4}$$

where *λ* is the friction coefficient or Darcy’s coefficient, and *V_0 _* is the velocity of liquid in the bendable tube. *P*_*j*1_ and *P*_*j*2_ can be calculated from:

(6) $$P_{j}=\frac{1}{2}\xi \rho v^{2},$$

where *v* is the local liquid velocity and *ζ* is the drag coefficient. Since the liquid velocities in the ampoule chamber as well as in the bendable tube are much smaller than injection velocity *V_1_*, and *ζ* is also very small (in the range of 0.03~0.1), *P*_*j*1_ and *P*_*j*2_ are neglected in the calculation. Thus, the following equation can be obtained from Equations (4) and (5).

(7) $$V_{1}=\sqrt{\frac{2\left(P-P_{G}\right)+\rho {\left(\frac{dX_{p}}{\mathrm{dt}}\right)}^{2}}{\rho_{0}+\frac{\lambda \rho L_{1}D_{1}^{4}}{D_{0}^{5}}}}$$

Substituting Equation (7) into Equation (3) will get:

(8) $$\frac{\mathrm{dP}}{\mathrm{dt}}=\frac{\left(P+B\right)\frac{dX_{p}}{\mathrm{dt}}-\frac{D_{1}^{2}}{D_{p}^{2}}B\sqrt{\frac{2\left(P-P_{G}\right)+\rho {\left(\frac{dX_{P}}{\mathrm{dt}}\right)}^{2}}{\rho_{0}+\frac{\lambda \rho D_{1}^{4}}{D_{0}^{5}}}}}{L-X_{P}+\frac{D_{0}^{2}}{D_{P}^{2}}L_{1}}$$

The strained energy loss of the bendable tube during injection *P_G_* can be obtained by strain energy *G* generated from elastic deformation over volume difference of deformation $\Delta V,\;i.e.\;P_{G}=\frac{G}{A\Delta d,}\;\mathrm{where}\;A=2\pi r_{1}L_{1}^{'}$ is the inner surface area and Δ*d*=2(*r*_1_ − *r*) is the diameter increment; and *r* and *r*_1_ are the inner radius of the bendable tube before and after deformation, *L*_1_^′^ is the length of bendable tube after deformation, as shown in Fig. (**4**). The strain energy *G* can be calculated by applying the generalized Hooke's Law, and using force and energy balance on an element of the bendable tube before accumulation. The following expression can be achieved (detailed explanation see [15]).

(9) $$G=\frac{\pi \left(R_{1}+r_{1}\right)r_{1}^{2}L_{1}^{'}P^{2}}{2E}\left[\frac{1}{\Delta }-\frac{2\mu r_{1}}{R_{1}^{2}-r_{1}^{2}}+\frac{\Delta r_{1}^{2}}{{\left(R_{1}^{2}-r_{1}^{2}\right)}^{2}}\right],$$

where *R*_1_ is the outer radius of the bendable tube after deformation, *δ* is the tube thickness, *R*_1_ = *r*_1_ + *δ*, as shown in (Fig. **4**). *μ* is the Poisson’s ratio, and *E* is the Young’s modulus of the bendable tube. According to [15], *L*_1_^′^ can be obtained:

(10) $$L_{1}^{'}=L_{1}\left[1+\frac{P}{E}\left(\frac{r^{2}}{R^{2}-r^{2}}-\frac{\mu r}{\Delta }\right)\right],$$

where *R* is the outer radius of the bendable tube before deformation, as shown in Fig. (**4**). *r*_1_ can be obtained:

(11) $$r_{1}=r\left(1+\frac{P}{E}\left(\frac{r}{\Delta }-\frac{\mu r^{2}}{R^{2}-r^{2}}\right)\right),$$

The strain energy loss *P_G_* thus can be calculated as:

(12) $$P_{G}=\frac{L_{1}^{'}P^{2}r_{1}^{2}\left(\frac{1}{\Delta }-\frac{2\mu r_{1}}{R_{1}^{2}-r_{1}^{2}}+\frac{\Delta r_{1}^{2}}{{\left(R_{1}^{2}-r_{1}^{2}\right)}^{2}}\right)}{4\pi r_{1}L_{1}^{'}\left(r_{1}-r\right)}$$

**Fig. (3):**
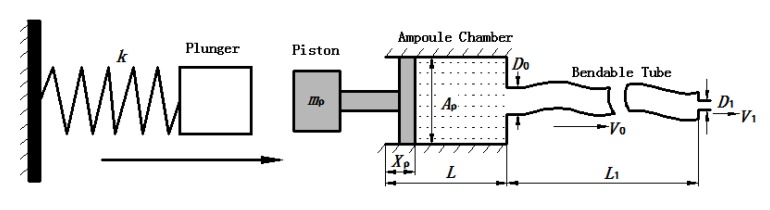
Physical model of split-type NFID powered by a spring.

**Fig. (4):**
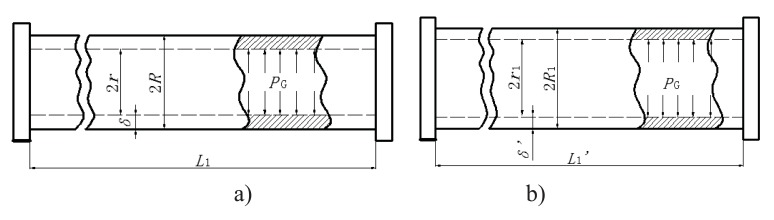
Indication of parameters of bendable tube: a) before deformation; b) after deformation.

The force balance equation for the driving piston is:

(13) $$m_{p}\cdot \frac{d^{2}X_{p}}{dt^{2}}=k\left(X_{0}-X_{p}\right)-A_{p}P-F_{f}$$

where *F_f_* is the resistant force exerted on the ring seal, *X*_0 _ is the initial compression length of the spring, and *m_p_* is the mass of the piston, as shown in (Fig. **3**).

The friction force can be achieved by the following expression, according to analysis of the same author [13]:

(14) $$F_{f}=\pi D_{p}b\theta P_{c}$$

where *θ* is the friction coefficient of the contact surface; *b* is the contact width between the piston and the chamber wall; *θ* ≈0.2 and b≈1 mm (measured data) are used in the calculation. *P_c_* is the pressure due to the ring compression in the absence of liquid. From finite element analysis, the value of *P_c_* is about 3.5MPa [13].

Thus, the mathematical model describing the injection pressure of a split typed and spring powered needle free injection system is expressed by:

(15) $$\left\{\begin{array}{c} \frac{\mathrm{dP}}{\mathrm{dt}}=\frac{\left(P+B\right)\frac{dX_{p}}{\mathrm{dt}}-\frac{D_{1}^{2}}{D_{p}^{2}}\sqrt[{B}]{\frac{2\left(P-P_{G}\right)+\rho {\left(\frac{dX_{p}}{\mathrm{dt}}\right)}^{2}}{\rho_{0}+\frac{\lambda \rho L_{1}D_{1}^{4}}{D_{0}^{5}}}}}{L-X_{p}+\frac{D_{0}^{2}}{D_{p}^{2}}L_{1}}\\ \frac{d^{2}X_{p}}{dt^{2}}=\frac{k\left(X_{0}-X_{P}\right)}{m_{p}}-\frac{A_{p}P}{m_{p}}-\frac{\pi D_{p}b\theta P_{c}}{m_{p}}\\ P_{G}=\frac{\left(R_{1}+r_{1}\right)L_{1}^{'}P^{2}r^{2}\left[\frac{1}{\Delta }-\frac{2\mu r}{R^{2}-r^{2}}+\frac{\Delta r^{2}}{{\left(R^{2}-r^{2}\right)}^{2}}\right]}{8Er_{1}L_{1}^{'}\left(r_{1}-r\right)} \end{array}\right.$$

Since the variation of liquid density is very small compared to other terms, *ρ* is simply change to *ρ*_0 _ for easy calculation. The local energy loss at the nozzle and the connectors and the energy loss from check valves are neglected in this model. The above equations can be decoupled and can be solved by Runge-Kutta integration method. In order to solve the equations, initial conditions need to be defined. In the spring-powered injection process, there is often a gap between the plunger and the piston before triggering, in order to ensure the strength of the impact. The width of the gap can influence the fluid flow and the dynamic pressure distribution. The initial velocity of fluid can be obtained by solving the momentum balance between the plunger and the combination of the piston and the fluid. In our calculation model, for simplicity, the piston is assumed to have an initial velocity to take into account of the plunger impacting effects, and its value is calibrated by the experimental value. If there is no impact gap, then an infinitesimal value is given to the initial velocity for easy calculation (10µm/s was used in calculation). Other initial settings are: the initial displacement of the piston is zero; the initial pressure of the fluid is atmosphere pressure.

In order to compare the calculated results to the experimental results, the parameters and settings used in calculation are the same as those used in experiments, and are listed in Table **1**. The self-made prototype has the injection volume 0-1ml, and the liquid volume of 0.3ml was chosen for calculation and experiments. The wire braid reinforced hydraulic rubber tube was chosen as the bendable tube for calculation and measurements, and its physical properties are described in German Standard DIN EN853 [16].

## 4 EXPERIMENTAL VERIFICATION AND CALCULATION RESULTS

In order to verify the accuracy of the mathematical model, a pressure measurement device was build as shown in Fig. (**5a**). A force transducer with frequency response of 0.1ms (FSG-15N1A, Honeywell, NJ, USA) was attached to the self-made platform as shown in Fig. (**5b**). The output end of the sensor is connected to a differential amplifier, and then transferred to a data collector (PCI-1711L, Advantech, Taiwan), and is shown on a computer. The handheld part of NFID (injector) is clamped and supported on the platform, and the nozzle of the injector is kept perpendicular to the force sensor. Knowing the nozzle diameter, the dynamic injection pressure can be calculated from the measured force [13]. Figures of experimental measured injection pressure can be obtained.

Fig. (**6**) shows both calculated and measured injection pressure vs. time starting from the point of injection (t=0), with the system variable listed in Table **1**. The profiles of the dynamic injection pressure within 100ms after injection are shown in Fig. (**6a**), while same profiles within 30ms after injection are shown in Fig. (**6b**). The injection pressure oscillates and gradually decreases in the following 10-100ms. The force balance equation (15) is a variation of the classic damped harmonic oscillator, and both calculated and measured results reflect the theoretical analysis. The glitches on the experimental profile are results of direct connection of the measured data without interpolation or data fitting. The maximum injection pressure occurs at the first peak of the oscillating curve. It is reported that the maximum injection pressure needs to reach a threshold of 14MPa before the jet is able to penetrate into human skin [17]. The predicted results generally agree to the experimental results, as the damping frequency and the peak values match. It is thus assumed that the prediction equations can be used to test and optimize various physical parameters of the split-typed needle-free injection system (for examples, straight vs. bendable tube; tube length; spring stiffness; type of tube material, *etc*.).

However, the oscillation behaviour of the measured injection pressure somehow lags behind the predicted results, which might be caused by the response delay of the transducer. In addition to that, there are discrepancies between calculated peak pressures and the experimental peak pressures. In particular, the calculated maximum pressure is about 15% larger than the measured maximum pressure. The estimated values such as friction coefficient, the pressure due to the ring compression, and some factors that are not included in the model such as the edge effects of the nozzle, may all contribute to the differences between predicted values and the measured values.

**Fig. (7):**
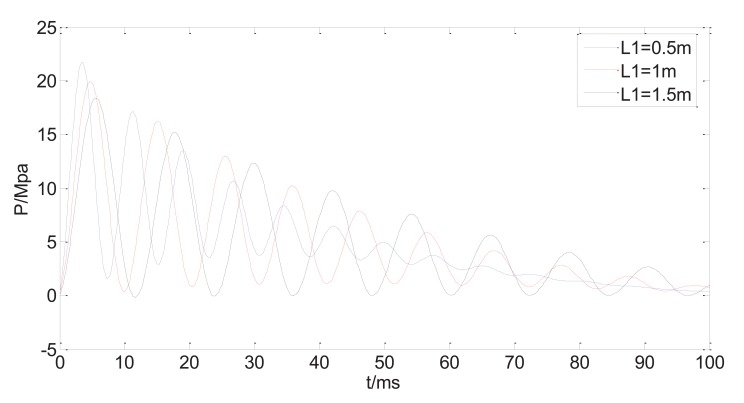
Predicted injection pressure with different length of bendable tube.

If the length of the bendable tube varies whereas other system variables are kept constant, the calculated injection pressure is shown in (Fig. **7**). The three curves of the dynamic pressure within 100ms correspond to tube length *L_1_=*0.5, 1, 1.5m, respectively. Obviously, the first peak of the dynamic pressure decreases as the tube length increases, and its occurrence time delays with increasing tube length. As the initial peak pressure has a dominant impact on jet penetration of the skin. The peak pressure needs to be above some threshold value for the jet to be able to piece into the skin. Also the peak pressure needs to be as close as possible to the injection time to avoid liquid splash. Thus, the shorter the bendable tube, the stronger the injection capability the NFID has. The increase of the tube length will also increase the injection time, resulting possible incomplete injection. From the design point of view, it is better to choose shorter bendable hose without scarifying the convenience of operation.

Fig. (**8**) shows the two curves of the dynamic injection pressure, corresponding to NFID with bendable tube (L1=0.5m), and NFID without bendable tube. It can be seen that the dynamic pressure curve without bendable tube has a much higher peak pressure which occurs instantly after injection time. The NFID without bendable tube thus has a much stronger penetration capability compared with the NFID with bendable tube. The elastic deformation of the bendable tube obviously functions as energy storage to slow down the rapid decaying and oscillated pressure.

The reduction of initial peak pressure with increasing tube length may be compensated with an increasing driving power. Fig. (**9**) shows three curves of the dynamic injection pressure, corresponding to three different spring stiffness, with other system parameters are kept the same as in Table **1**. Apparently, the increasing driving force corresponding to higher spring stiffness will increase the peak pressure without affecting the occurrence time of the peak pressure.

Fig. (**10**) shows four curves of the dynamic injection pressure, corresponding to four different bendable tube materials, with other system variables kept same as Table **1**. It can be seen from Fig. (**10**) that the peak pressure varies with the material property of the bendable tube. The wire braid reinforced hydraulic rubber tube (*E*=5×10^7^, *μ*=0.4) has the largest peak injection pressure, which is weaker for rubber tube (*E*=8×10^6^, *μ*=0.47 [18],), and the silicone tubes 1(*E*=5.7×10^6^, *μ*=0.5) and 2(*E*=2.6×10^6^, *μ*=0.49) have minimum peak pressure [19]. The peak pressure increases with the increasing Young’s modulus of the bendable tube, and is sensitive at smaller values of Young’s modulus. The peak pressure does not increase much even Young’s modulus increases much at larger values. Apparently, the different tube materials only vary the peak pressure without affecting the occurrence time of the peak pressure.

## 5 *IN VIVO* TEST

A preliminary *in vivo* test was carried out to indicate the effects of the maximum injection pressure on animal injection. The self designed NFID without bendable tube was used for the test. Future *in vivo* tests will be carried out to include the effects of the bendable tube length and the materials. A 20 day age Duroc piglet provided by Liuhe Animal Science Base of Jiangsu Academy of Agricultural Sciences (China) was selected for injection of blue dyed saline. Four different springs with increasing spring stiffness were applied for different driving forces. The dynamic injection pressure was measured by aforementioned pressure measurement apparatus and its maximum injection pressure was recorded, respectively. The piglet was captured and its back neck and lateral parts was pushed against the nozzle of NFID for each injection of 1ml dose, respectively. The sites to be injected were separated by a minimum of 5 cm, The piglet was killed and the injection section was segmented for analysis of the amount of intramuscular drug deposition, residual skin dose, and local site reaction.

Fig. (**11**) shows the diffusion of dyed saline in subcutaneous and intramuscular tissue of the piglet under four different maximum injection pressures. Under four injection pressure conditions, the dyed saline can all penetrate into the subcutaneous region and little residual occurs on the injection spot. The dyed saline can only reach subcutaneous layer instead of intramuscular layer for the case of maximum injection pressure of 24Mpa. The dye can diffuse into the intramuscular layer for the cases of injection pressure of 32, 40, and 48Mpa. The injection depth and the proliferation of dye diffusion increase with the increasing injection pressure, as expected. The *in vivo* test again confirms the injection pressure is a dominant factor for the jet performance of NFID. In addition to the value of the maximum injection pressure, the growth rate of the injection pressure may also affects the jet performance of NFID, including the rate of penetration of drugs that affected the pain.

## CONCLUSION

A continuous needle-free injection device (NFID) was designed for animal vaccination. The device is driven by spring force and has the feature of split type. A mathematical model was presented to calculate the dynamic injection pressure based on the current designed split typed NFID. The strain energy loss and the lengthwise pressure loss of the flexible hose were implemented into the model. An experimental setup was build to measure the dynamic injection pressure of the designed split typed NFID. Under the same system conditions, the calculation results of the dynamic injection pressure match the experimental results.

It is found from the calculation that the bendable tube of the split typed NFID has significant impact on the profile of the injection pressure. The initial peak pressure is less than the initial peak pressure of NFID without bendable tube, and there is occurrence time lag of the peak pressure compared to that of NFID without bendable tube. The longer the bendable tube, the less the peak pressure, and the longer the occurrence delay time. Since the profile of the injection pressure, particularly the peak pressure, determines the jet injection capability, the bendable tube will reduce the jet injection capability, and may cause splashing or incomplete injection. The reduced peak pressure caused by the bendable tube can be compensated by increasing the driving force, or by increasing spring stiffness for the current NFID. However, the increasing driving force has little influence on the time delay of the peak pressure occurrence. The different material properties of bendable tube, particular Young’s Modulus, have influences on peak injection pressure, but have little effects on occurrence time. The mathematical model and the calculation results provide useful guidance in optimization of the system design of the current NFID.

Although NFID for animal vaccination has been existed for decades, using needle-free technology for vaccination of large animals still presents a challenging work. The presented NFID prototype applies hydraulic power driving by a spring force with simple mechanical structure. The mathematical model proposed in the current study is the first attempt to implement the strain energy loss and pressure loss in the bendable tube, and reveal the relationship between the injection pressure and the system variables of split typed NFID. The model provides engineering guidance for optimization of the current type NFID. The current model is also applicable to other driving forces such as magnetic forces, as long as the physical behaviour of the driving force can be described mathematically.

## Figures and Tables

**Fig. (5):**
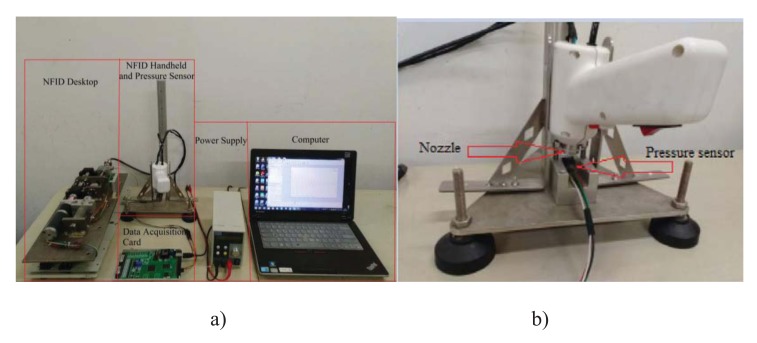
Injection pressure measurement setup: a) whole system; and b) close-up view of the pressure sensor.

**Fig. (6a):**
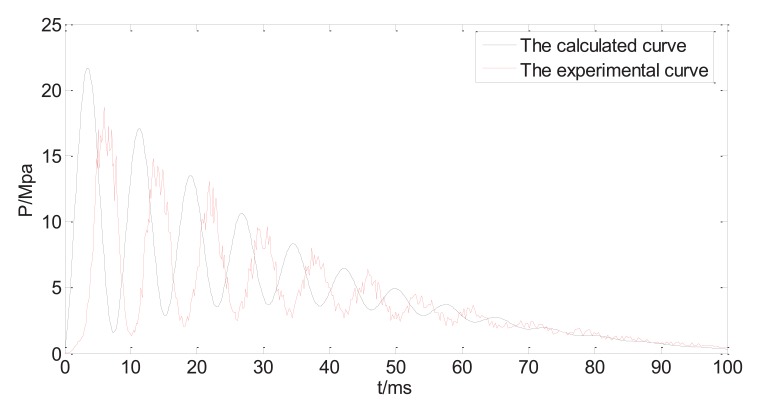
Measured and predicted injection pressure (first 100ms).

**Fig. (6b):**
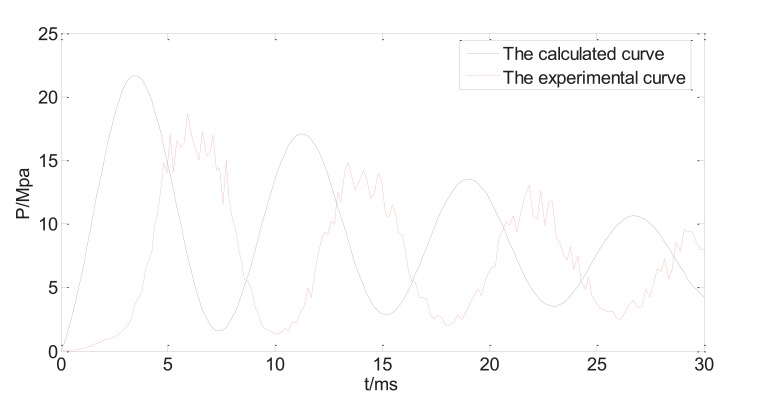
Measured and predicted injection pressure (first 30ms).

**Fig. (8):**
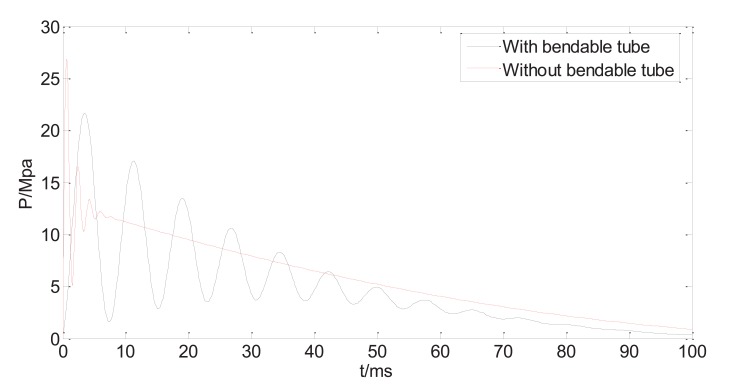
Predicted injection pressure from NIFD with/without bendable tube.

**Fig. (9):**
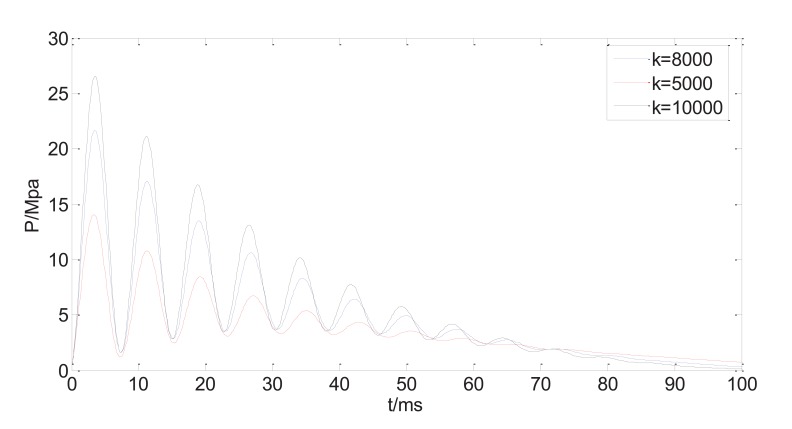
Predicted injection pressure with different spring stiffness.

**Fig. (10):**
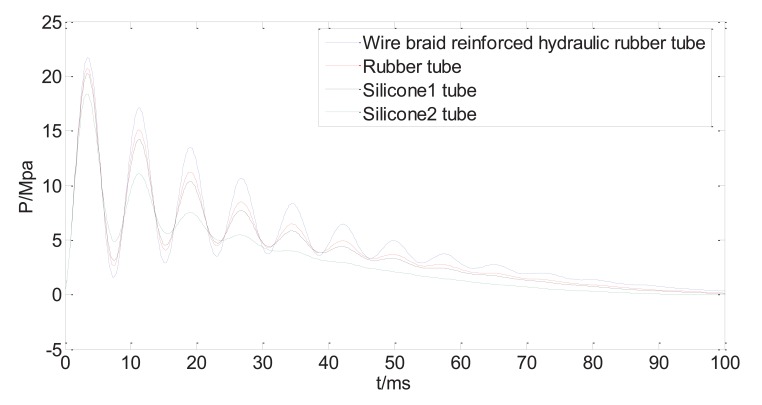
Predicted injection pressure with different bendable tube materials.

**Fig. (11):**
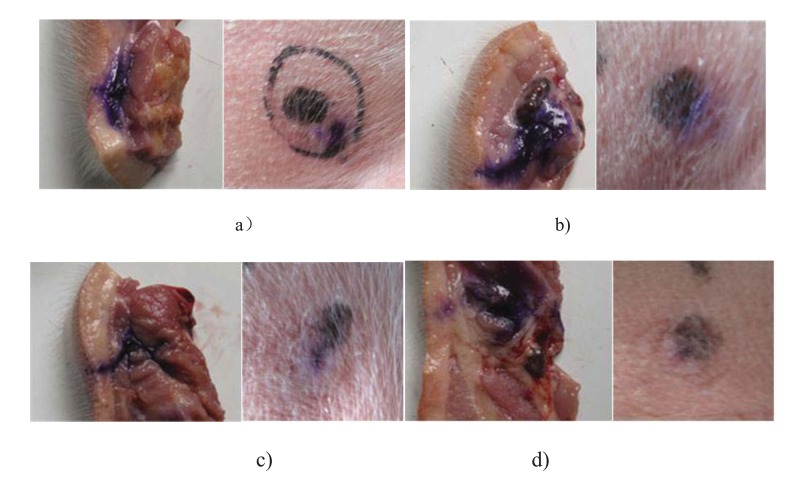
Dyed saline diffusion under the skin (left) and the outlook of injection spot (right) under four different injection pressures: a) 24MPa; b) 32MPa; c) 40MPa; d): 48MPa.

**Table 1:** System parameters for calculation and measurements.

No.	Parameter (Unit)	Value
1	Bulk modulus of fluid *B* (Pa)	2.18×10^9^
2	Gravity acceleration g(m/s^2^)	9.8
3	Friction coefficient *λ*	0.1
4	Fluid Density *ρ*_0 _(kg/m^3^)	998
5	Effective length of chamber *L*(m)	0.028
6	Compression length of spring *X*_0 _(m)	0.031
7	Stiffness of spring *k*(N/m)	8000
9	Nozzle diameter *D*_1_(m)	2.5×10^-4^
10	Diameter of chamber *D_p_*(m)	4.8×10^-3^
11	Mass of piston *m_p_*(kg)	0.12
12	Inner diameter of bendable tube *D*_0 _(m)	0.005
13	Outer radius of bendable tube *R*(m)	0.006
14	Length of bendable tube *L*_1_(m)	0.5
15	Contact width *b*(m)	1×10^-3^
16	Poisson’s ratio *μ*	0.4
17	Young’s modulus of bendable tube *E*(Pa)	5×10^7^
18	Friction coefficient of contact surface *θ*	0.2
